# Extracellular ATP is involved in dsRNA-induced MUC5AC production via P2Y2R in human airway epithelium

**DOI:** 10.1186/s12931-016-0438-0

**Published:** 2016-09-27

**Authors:** Yutaka Shishikura, Akira Koarai, Hiroyuki Aizawa, Mutsuo Yamaya, Hisatoshi Sugiura, Mika Watanabe, Yuichiro Hashimoto, Tadahisa Numakura, Tomonori Makiguti, Kyoko Abe, Mituhiro Yamada, Toshiaki Kikuchi, Yasushi Hoshikawa, Yoshinori Okada, Masakazu Ichinose

**Affiliations:** 1 Department of Respiratory Medicine, Tohoku University Graduate School of Medicine, 1-1 Seiryo-machi, Aoba-ku, Sendai 980-8574 Japan; 2 Department of Advanced Preventive Medicine for Infectious Disease Tohoku University Graduate School of Medicine, 1-1 Seiryo-machi, Aoba-ku, Sendai 980-8575 Japan; 3 Department of Pathology, Tohoku University Hospital, 1-1 Seiryo-machi, Aoba-ku, Sendai 980-8574 Japan; 4 Department of Respiratory Medicine and Infectious Diseases, Niigata University Graduate School of Medical and Dental Sciences, 1-757 Asahimachidori, Chuo-ku, Niigata 951-8510 Japan; 5 Department of Thoracic Surgery, Fujita Health University School of Medicine, Toyoake, 470-1192 Japan; 6 Department of Thoracic Surgery Institute of Development, Aging and Cancer Tohoku University, 4-1 Seiryo-machi, Aoba-ku, Sendai 980-8575 Japan

**Keywords:** COPD, Exacerbation, Mucin, Pannexin channel, Viral infection

## Abstract

**Background:**

In response to tissue damage or inflammation, adenosine-5′-triphosphate (ATP) is released into the extracellular compartment and has been demonstrated to augment inflammation via purinergic P2 receptors (P2Rs). Recently, ATP has been shown to be increased in the airways of COPD patients. In the present study, we examined the possible involvement of extracellular ATP in airway mucus hypersecretion during viral-induced COPD exacerbations.

**Methods:**

The involvement of extracellular ATP in the release of a major airway mucin, MUC5AC, and its signal pathway was examined after stimulation with polyinosine-polycytidylic acid [poly(I:C)], a synthetic analog of dsRNA to mimic viral infection, and rhinovirus (RV) infection in NCI-H292 cells and differentiated airway epithelial cells from COPD patients.

**Results:**

Treatment with poly(I:C) significantly increased the amount of extracellular ATP and induced MUC5AC release in NCI-H292 cells. Pre-treatment with a pannexin channel inhibitor, carbenoxolone (CBX), reduced the amount of extracellular ATP and suppressed MUC5AC release from poly(I:C)-treated cells. Pre-treatment with the P2R antagonist suramin significantly reduced the expression and release of MUC5AC. The inhibitory effects of CBX and suramin on the release of ATP and/or MUC5AC were replicated with RV infection. Pre-treatment with suramin also significantly reduced the expression and amount of extracellular EGFR ligands and the phosphorylation of EGFR and ERK in poly(I:C)-treated cells. In addition, pre-treatment with a P2Y2 receptor siRNA significantly suppressed the poly(I:C)-potentiated EGFR ligands and MUC5AC release. After poly(I:C) stimulation, the expression of MUC5AC in the differentiated cells from COPD patients was significantly higher than those from healthy subjects and the values of MUC5AC expression were inversely related with forced expiratory volume in 1 s (FEV1) % predicted. The inhibitory effects of CBX and suramin on poly(I:C)-potentiated MUC5AC expression were confirmed in differentiated airway epithelium from COPD patients.

**Conclusions:**

These results demonstrate that dsRNA induces the release of ATP via pannexin channel and that the extracellular ATP is involved in the expression and release of MUC5AC, mainly via P2Y2R, in an autocrine manner. Modulation of this pathway could be a therapeutic target for viral-induced mucus hypersecretion in COPD exacerbations.

## Background

Chronic obstructive pulmonary disease (COPD) is a leading cause of morbidity and mortality throughout the world [1]. Exacerbations of COPD, defined as worsening of the symptoms including cough, sputum, and/or dyspnea, cause an acute deterioration in airway inflammation and lung function, and it also leads an acceleration of the rate of decline of lung function and increasing the mortality [2, 3]. Therefore, preventing exacerbations of COPD is necessary. However, the precise mechanisms by which exacerbations occur have not been fully elucidated.

Adenosine-5′-triphosphate (ATP) is well known as an energy source in the intracellular compartments. On the other hand, once released into the extracellular compartment in response to tissue damage or inflammation, extracellular ATP has been shown to act as a potent inducer of inflammation [4, 5]. Although ATP is known to be released from necrotic cells [6], it has been recently demonstrated to be released from intact cells through pannexin channels [7–9]. Extracellular ATP acts via purinergic P2 receptors (P2Rs) composed by seven members of the P2X receptor (P2XR; P2X1R-P2X7R) and eight members of the P2Y receptor (P2YR; P2Y1R, P2Y2R, P2Y4R, P2Y6R, P2Y11R-P2Y14R); the former are ligand-gated ion channels and the latter are G protein-coupled receptors [4, 5]. Especially, P2X7R has been shown to be involved in inflammation [10], and P2Y2R reported to be involved in the release of mucin from secretory glands [11, 12]. In the airway of COPD patients, an increased concentration of extracellular ATP has been demonstrated, especially during acute smoke exposure [13], suggesting that extracellular ATP might be involved in the pathophysiology of COPD and exacerbations.

Mucus hypersecretion in the airway is a common feature of this disease, especially during exacerbations. Although mucin production is a component of the innate immune defense mediated by airway epithelia, mucus overproduction causes airway obstruction, which may lead to COPD exacerbations [14–16]. Viral infection, which is known as the primary cause of COPD exacerbations, induces mucus secretion [17] and the production of a major secreted mucin, MUC5AC [18]. Viral-induced immune reactions are mediated by Toll-like receptors (TLRs), especially TLR3, through the recognition of viral-derived double-stranded RNA (dsRNA) [19, 20]. Recently, we and others have shown that viral infection and the stimulation of TLR3 induces the expression and production of MUC5AC in airway epithelial cells mainly via epidermal growth factor receptor (EGFR) and extracellular signal-regulated kinase (ERK) signaling pathways [21, 22].

Viral infection and dsRNA have been shown to increase the amount of extracellular ATP [23, 24] and the production of mucin in airways via TLR3 [21, 22]. However, the involvement of extracellular ATP in viral-induced mucin production in airway epithelial cells remains unclear. In the present study, we evaluated the following points, using NCI-H292 cells and differentiated primary human bronchial epithelial cells (HBECs) from normal subjects and COPD patients: (a) whether pannexin channels are involved in ATP release, (b) whether the inhibition of P2Rs, especially P2X7R and P2Y2R, affects MUC5AC production and the EGFR-ERK signaling pathway after stimulation with a synthetic dsRNA analogue, polyinosinic-polycytidylic acid [poly(I:C)], as a TLR3 ligand to mimic viral infection, and rhinovirus infection.

## Methods

### Materials

Poly(I:C) (polyinosinic acid/polycytidylic acid, sodium salt, double-stranded) were purchased from Calbiochem (La Jolla, CA). Carbenoxolone (CBX), suramin, Brilliant Blue G (BBG) and A438079 were from Sigma-Aldrich (St. Louis, MO).

### Patients

Five control subjects including four never smokers and one ex-smoker without COPD and five former smokers with COPD took part in our study after giving written informed consent (Table 1). COPD was diagnosed according to the GOLD guidelines [1]. All subjects had undergone surgery for lung cancer after receiving pulmonary function tests. Human bronchial tissues were obtained from the 2-4 bronchi of the lobe resected at surgery, avoiding areas involved by tumors. The tissues were used for the culture of human bronchial cells. All experiments in the study were approved by ethics committee of Tohoku University Graduate School of Medicine.

**Table 1:** Clinical characteristics of patients

	Healthy subjects (*n* = 5)	COPD (*n* = 5)
Sex (Male/Female)	1/4	5/0
Age (years)	69.5 ± 6.06	70.6 ± 3.84
Ex-smoker	1	5
smoking history (pack-year)	8.0 ± 17.9	76.7 ± 73.7*
FVC (L)	2.82 ± 0.24	3.30 ± 0.38*
FVC % predicted	115 ± 12.3	96.0 ± 7.89*
FEV_1_ (L)	2.18 ± 0.15	2.14 ± 0.50
FEV_1_ % predicted	113 ± 19.2	74.3 ± 17.0*
FEV_1_/FVC (%)	77.5 ± 9.54	64.0 ± 10.2
DLCO/VA % predicted	102 ± 10.5	86.2 ± 8.62*

*COPD* chronic obstructive pulmonary disease; pack-year: 1 year smoking 20 cigarettes-day; *FVC* forced vital capacity; *FEV1* forced expiratory volume in one second; *DLCO* diffusing capacity of the lung for carbon monoxide; *VA* alveolar volume. Values are mean ± SE. *p < 0.05 compared to healthy subjects

### Preparation of epithelial cells

NCI-H292 cells, a human pulmonary mucoepidermoid carcinoma cell line (ATCC, Manassas, VA) were cultured according to our previous study [22]. Cells were cultured in RPMI-1640 medium supplemented with 10 % FBS at 37 °C in a humidified atmosphere of 5 % CO_2_. Cells were grown to 80 % confluence in 24-well and maintained in FBS-free medium for 24 h before stimulation. Primary human bronchial epithelial cells (HBECs) obtained from lobes resected from patients at surgery were used for the air–liquid culture at passages 2–3. The air-liquid culture of HBECs was conducted using Clonetics® B-ALI^TM^ Air-Liquid Interface (ALI) according to our previous study [22]. The ALI state was maintained for 7–10 days, as previous studies have shown this duration is required for mucociliary differentiation [25, 26].

To investigate the effect of poly(I:C) on the cells, supernatants were harvested at 24 h after treatment with poly(I:C) at the concentration of 10 μg/ml [22]. A pannexin channel inhibitor, CBX, a non-selective P2R antagonist, suramin, a non-selective P2X7 antagonist, BBG and a selective P2X7 antagonist, A438079 were added to the media at various concentrations 30–60 min prior to poly(I:C)-treatment. To stimulate ALI state cells, 100 μl of media containing poly(I:C) was added on top of cells at the apical side of differentiated epithelial cells.

### Rhinovirus infection

A stock solution of type 14 rhinovirus (RV14) [1.0 × 10^7^ tissue culture infectious dose (TCID_50_)/ ml] was prepared from a patient with a common cold and the rate of RV14 release was titrated according to previously described methods [27]. NCI-H292 cells cultured in 24-well plates were infected with RV14 at a multiplicity of infection (MOI) of 1 for 90 min in RPMI-1640 medium at 33 °C before the virus was removed and replaced with RPMI-1640 medium [18, 28]. In some experiments, cells were pretreated with CBX or suramin 30–60 min prior to infection. After the cells were cultured for 24–48 h at 33 °C, the supernatants were removed and stored at −80 °C until required.

### Measurement of ATP

To measure the amount of ATP in cell culture supernatants, samples were centrifuged at ice-cold at 14,000 rpm for 2 min and the supernatants were stored at −80 °C prior to analysis. The amount of ATP was measured using ATP Assay Kit (BioVision Inc.; Milpitas, CA) according to the manufacturer’s instructions.

### ELISA

MUC5AC protein was measured by ELISA based methods according to our previous study [21]. Amphiregulin and TGF-α were measured using ELISA (R&D Systems, Inc.; Minneapolis, MN) according to the manufacturer’s instructions.

### Immunocytochemistry

Cells were seeded in 8-well chamber slides at a density of 1 × 10^5^/ml and cultured for 24 h, and then the medium was replaced with FBS-free medium for a further 24 h. After washing with PBS, the slides were fixed with freshly prepared 4 % paraformaldehyde in PBS for 10 min at room temperature. The slides were permeabilized with 0.1 % Triron X-100 in PBS for 10 min at room temperature. The slides were then blocked for 30 min at room temperature by 1 % skim milk, rinsed, and then incubated overnight with mouse monoclonal anti-MUC5AC (Clone 45 M1) antibody (1:50 dilution; Thermo Fisher Scientific, Fremont, CA) at 4 °C. After washing with PBS, the slides were incubated with goat anti-mouse IgG conjugated with FITC (1:200 dilution; Abcam, Cambridge, UK) for 1 h at room temperature. After washing, the nuclei of the cells were stained with Fluoromount-G containing DAPI (Southern Biotech, Birmingham, AL). The slides were then viewed with a multiphoton confocal LSM 780 NLO microscope system (Carl Zeiss, Jena, Germany) under x400 magnification.

### Immunoblotting

Cells were seeded in 6-well or 60 mm dishes at a density of 1 × 10^5^/ml. At 80 % confluence, cells were maintained in FBS-free medium for 24 h before stimulation. To evaluate the inhibitory effect of suramin on the expression of phospho-EGFR (pEGFR) and phospho-extracellular signal-regulated kinase (pERK), the cells were treated with suramin 30 min prior to poly(I:C)-stimulation. Four hours after poly(I:C)-stimulation, cells were washed with ice-cold HANK’s balanced salt solution (HBSS) and homogenized in cell lysis buffer (0.05 % TritonX, 35 mM Tris-HCl, pH 7.4, 0.4 mM EGTA, 10 mM MgCl_2_, 1 μM phenylmethylsulfonyl fluoride, 100 μg/ml aprotinin and 1 μg/ml leupeptin) at 4 °C. Samples were solubilized in SDS-PAGE sample buffer. Equal amounts of protein were loaded and separated by electrophoresis on 12.5 % SDS polyacrylamide gels. After electrophoresis, separated proteins were transferred to a PVDF membrane (Bio-Rad Laboratories, Herculer, CA). Rabbit polyclonal anti-phospho-EGF receptor antibody (1:1000 dilution), rabbit polyclonal anti-EGF receptor antibody (1:5000), rabbit polyclonal anti-phospho-ERK1/2 antibody (1:1000), rabbit polyclonal anti-ERK1/2 antibody (1:5000, all from Cell Signaling Technology, Danvers, MA) were used to detect the target proteins. Appropriate peroxidase-conjugated secondary antibodies were detected using ECL-plus (Amersham Biosciences, Buckinghamshire, UK) and visualized with a chemiluminescence imaging system (LAS-4000 mini; Fujifilm, Tokyo, Japan). Each band intensity was quantified by densitometry (Quantity One software, Bio-Rad, Hercules, CA, USA).

### Real-time PCR

Total RNA was isolated from NCI-H292 cells and HBECs differentiated in ALI to prepare cDNA using TaqMan Gene Expression Cell-to-CT^TM^ Kit (Applied Biosystems, Foster City, CA) according to the manufacturer’s instructions. cDNA was amplified with specific primers to *MUC5AC*, *TGF-α*, *amphiregulin*, *TLR3* and *GAPDH* using the 7500 Real-Time PCR System (Applied Biosystems). Primers used to amplify cDNA were from TaqMan Gene Expression Assays (Biosystems): *MUC5AC* (catalogue number Hs01365616_ml), *TLR3* (Hs00152933_ml), *TGF-*α (Hs00608187_ml), *amphiregulin* (Hs00950669_ml) and *GAPDH* (Hs99999905_ml). Relative quantification of different transcripts was determined with the comparative threshold cycle (C_T_) method using *GAPDH* as the endogenous control. Results were calculated as fold induction over control.

### Silencing of P2R

Cells were seeded in 6-well plates in complete media. At 50 % confluence, transfection with siRNA was performed. In one tube, 9 μl of Lipofectamine RNAiMAX (Invitrogen Life Technologies, Grand Island, NY) were mixed gently with 150 μl Opti-MEM medium (Invitrogen Life Technologies, Grand Island, NY). In a separate tube, 300 pmol of non-targeting, *P2X7R or P2Y2R* siRNA (ON-TARGETplus SMARTpool; Dharmacon, Lafayette, CO) were mixed gently with 150 μl Opti-MEM medium. These siRNA and lipofectamine solutions were then combined, gently mixed and incubated for 5 min at room temperature. After incubation, the siRNA duplex-lipofectamine complexes were added to each dish (final concentration of siRNAs = 100 nM). Cells were incubated at 37 °C for 6 h. Then, media were changed and further cultured for 24 h. After that, media were changed to serum-free medium and further cultured for 24 h. The siRNA treated cells were then used to assess the role of the P2X7R or P2Y2R on MUC5AC release in the presence of 10 μg/ml poly(I:C) or to assess P2X7R or P2Y2R expression by immunoblotting.

### Cell viability assay

One mg/ml 3-(4,5-dimethylthiazol-2-yl)-2,5-diphenylterazolium bromide (MTT; Sigma-Aldrich, St. Louis, MO) was prepared using HBSS. Supernatants were removed from cells, and MTT solution was added to each well. After 1 h incubation at 37 °C, the MTT solution was discarded and DMSO was added to each well. The product was quantified at 570 nm with a micro plate reader. To evaluate the cell viability in ALI state cells, the lactate dehydrogenase (LDH) activity was measured in the supernatants from the cells using Cytotoxicity Detection Kit^PLUS^ (LDH) (Sigma-Aldrich) according to the manufacturer’s instructions.

### Statistical Analysis

Data are expressed as the mean ± SEM. GraphPad Prism (GraphPad Software Inc., SanDiego, CA) was used for statistical test. Experiments with multiple comparisons were evaluated using one way analysis of variance (ANOVA) by Bonferroni’s test to adjust for multiple comparisons. Mann-Whitney *U* test was used for single comparison. Spearman’s correlation was also used to assess statistical significance when applicable. Significance was defined as *p* < 0.05.

## Results

### Involvement of extracellular ATP on MUC5AC release in poly(I:C)-treated NCI-H292 cells

**Fig. 1:**
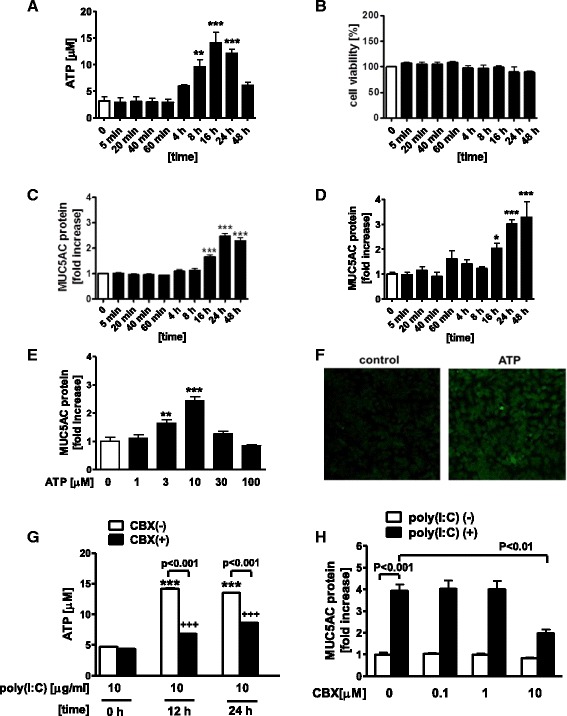
Involvement of extracellular ATP in MUC5AC release in poly(I:C)-treated NCI-H292 cells. **a**-**c** Effect of poly(I:C) on cell viability and the release of extracellular ATP and MUC5AC from NCI-H292 cells. Cells were treated with 10 μg/ml poly(I:C). At various time points after the incubation, whole cells and supernatants were harvested and assayed. **a** Supernatants were assessed for the concentration of extracellular ATP by fluorometric assay. **b** Cell viability was assessed by MTT assay. Cell viability was calculated as the percentage of viable cells among poly(I:C)-treated cells at 0 min. **c** Supernatants were assayed for MUC5AC release by ELISA. (D-E) Release of MUC5AC in ATP-treated cells. **d** Cells were treated with 10 μM ATP. At various time points after the incubation, the supernatants were harvested and assayed. **e** Cells were treated with various concentrations of ATP. After 24 h, the supernatants were harvested and assayed for MUC5AC release. **f** Panels show representative photographs of the immunoreactivity of MUC5AC in NCI-H292 cells. Treatment with 10 μM ATP augmented the immunoreactivity of MUC5AC. Original magnification: x200. **g**, **h** Effect of a pannexin channel inhibitor, carbenoxolone (CBX), on the release of extracellular ATP and MUC5AC in poly(I:C)-treated cells. **g** Cells were treated with 10 μM CBX or vehicle 1 h prior to the treatment with 10 μg/ml poly(I:C). At various points after incubation, the supernatants were harvested and assessed for the concentration of extracellular ATP. **h** Various concentrations of CBX were added 1 h before 10 μg/ml poly(I:C) treatment. After 24 h, the supernatants were harvested and assayed for MUC5AC release. The data are expressed as the means ± SEM for three to six separate experiments. *p < 0.05, **p < 0.01, ***p < 0.001 compared with the values of vehicle-treated control. ^+++^ *p* < 0.001 compared with the values of poly(I:C)-treated control

In our previous study, we confirmed that TLR3 was expressed in NCI-H292 cells, a human pulmonary mucoepidermoid carcinoma cell line, and a synthetic dsRNA analogue, poly(I:C), used as a TLR3 ligand to mimic viral infection, induced the expression and release of MUC5AC from the cells [22]. Here, to determine the involvement of extracellular ATP on MUC5AC release from airway epithelial cells after TLR3 stimulation, we investigated the amount of extracellular ATP in poly(I:C)-treated NCI-H292 cells. Treatment with poly(I:C) significantly increased the amount of extracellular ATP at 8 h or later at a concentration of 10 μg/ml (3.12 ± 0.80 at 0 h vs 9.56 ± 1.38 μM at 8 h, *p* < 0.001), without affecting cell viability (Fig. 1a and b). In the same way, poly(I:C)-potentiated MUC5AC release was shown at 16 h or later (at 24 h, 2.48-fold increase, *p* < 0.001) (Fig. 1c). Exogenous ATP alone also increased the release of MUC5AC at 16 h or later at a concentration of 10 μM (at 24 h, 3.01-fold increase, *p* < 0.001) (Fig. 1d). After 24 h stimulation with exogenous ATP, the release of MUC5AC was dose-dependently increased at a concentration of less than 10 μM (Fig. 1e) and the intracellular production of MUC5AC was confirmed by immunocytochemistry (Fig. 1f). Recently, cigarette smoke-bubbled medium was reported to induce the release of ATP from HBECs via pannexin channel [7], which suggested the possible involvement of pannexin channel in poly(I:C)-induced ATP release. Therefore, we investigated the effect of pannexin channel inhibitor, carbenoxolone (CBX) on the release of ATP from NCI-H292 cells after poly(I:C) stimulation. Pre-treatment with 10 μM CBX significantly reduced the amount of extracellular ATP in poly(I:C)-treated cells at 12 h and 24 h (at 12 h, 14.2 ± 0.29 vs 6.84 ± 0.21 μM, *p* < 0.001) (Fig. 1g). Pre-treatment of 10 μM CBX also partially but significantly suppressed poly(I:C)-induced MUC5AC release from NCI-H292 cells (*p* < 0.01) (Fig. 1h).

### Effect of P2R inhibition on MUC5AC production and release in poly(I:C)-treated NCI-H292 cells

**Fig. 2:**
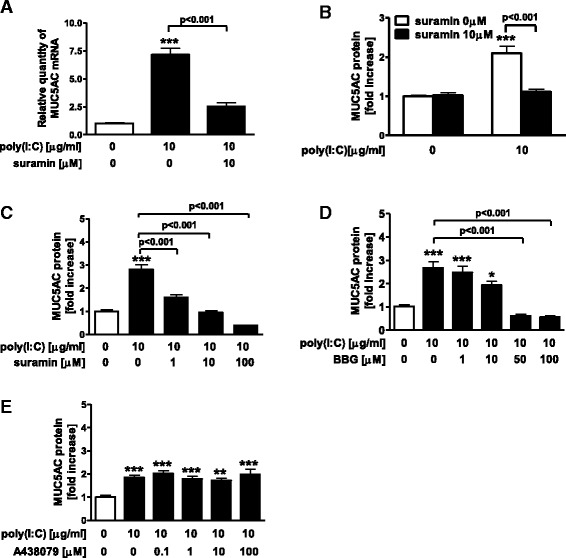
Effect of P2R antagonists on MUC5AC production and release in poly(I:C)-treated NCI-H292 cells. **a**-**c** Effect of a non-selective antagonist of P2R, suramin, on MUC5AC release in poly(I:C)-treated cells. Cells were treated with 10 μM suramin or vehicle 30 min prior to the treatment with 10 μg/ml poly(I:C). After 24 h, whole cells were harvested, and the expression of MUC5AC was assessed with Real-time PCR (**a**), and the intracellular protein of MUC5AC was assayed by ELISA (**b**). **c** Various concentrations of suramin were added 30 min before 10 μg/ml poly(I:C) treatment. After 24 h, the supernatants were harvested and assayed for MUC5AC release by ELISA. **d**, **e** Effect of P2X7R antagonists on MUC5AC release in poly(I:C)-treated cells. Various concentrations of a P2X7R antagonist, Brilliant Blue G (BBG), or a specific P2X7R antagonist, A438079, were added 30 min before 10 μg/ml poly(I:C) treatment. After 24 h, supernatants were harvested and assayed for MUC5AC release. The data are expressed as the means ± SEM for three to six separate experiments. *p < 0.05, **p < 0.01, ***p < 0.001 compared with the values of control

**Fig. 3:**
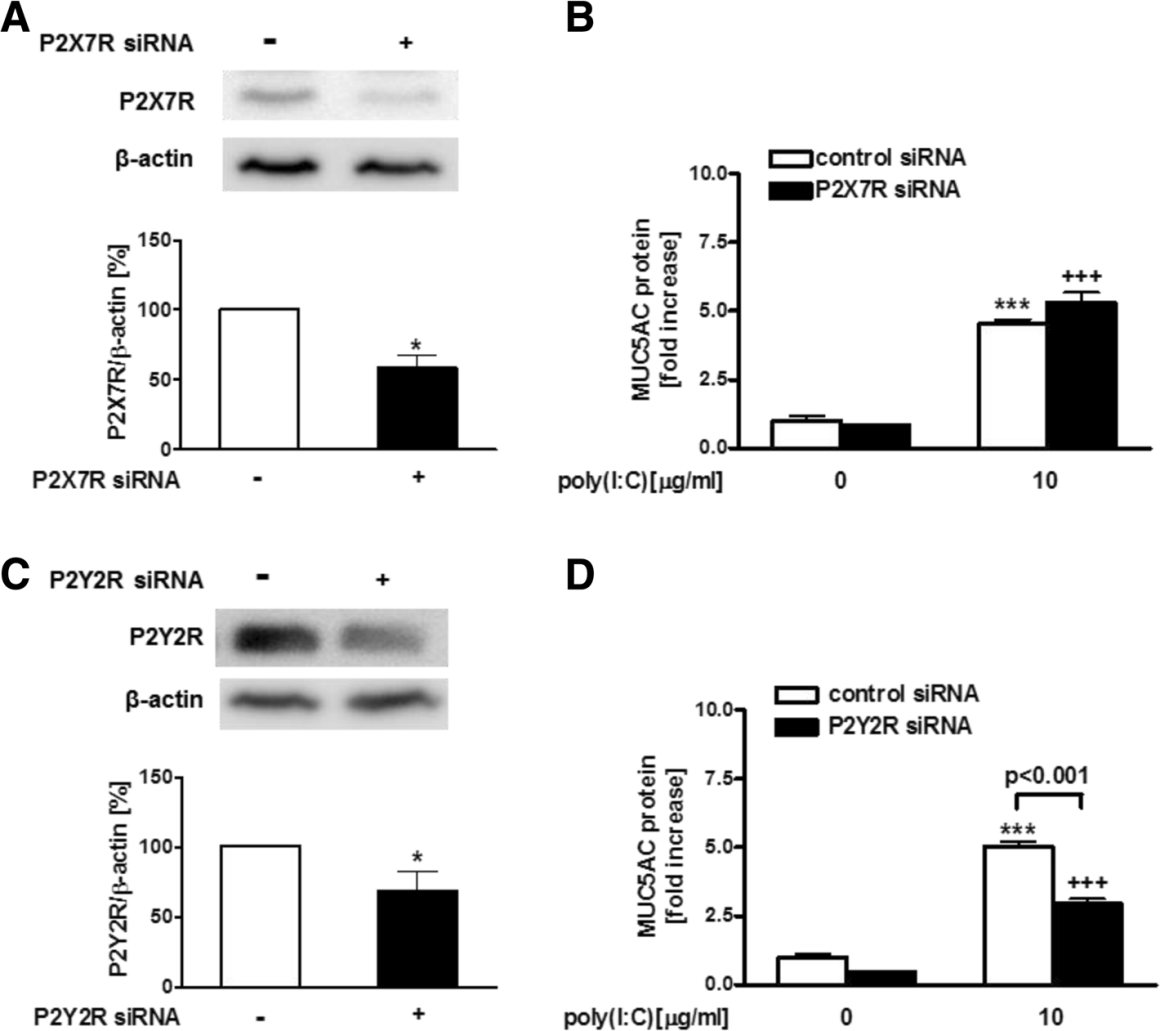
Effect of P2X7R or P2Y2R silencing with siRNA on MUC5AC release in poly(I:C)-treated NCI-H292 cells. **a**, **c** Effect of P2X7R or P2Y2R silencing with siRNA in NCI-H292 cells was evaluated by immunoblotting for 24 h after transfection. The amount of P2X7R or P2Y2R was assessed by densitometry. **b**, **d** Effect of P2X7R or P2Y2R silencing with siRNA on the release of MUC5AC in poly(I:C)-treated cells. Cells were treated with non-targeting, P2X7R or P2Y2R siRNA 48 h prior to the treatment with 10 μg/ml poly(I:C) or vehicle. After 24 h, supernatants were harvested and assayed for MUC5AC release by ELISA. The data were normalized by the values to control siRNA at 0 μg/ml poly (I:C). The data are expressed as the mean ± SEM for three to four separate experiments. P2X7 siRNA(-), P2Y2R siRNA(-) or control siRNA denotes non-targeting siRNA treatment. *p < 0.05, ***p < 0.001 compared with the values of control. ^+++^ *p* < 0.001 compared with the values of control siRNA

To investigate the effect of P2R antagonist on poly(I:C)-induced MUC5AC release in NCI-H292 cells, we used a non-selective antagonist of P2R, suramin. Suramin significantly inhibited the expression and production of poly(I:C)-potentiated MUC5AC at 10 μM (Fig. 2a, b) and also decreased the release of poly(I:C)-potentiated MUC5AC at the concentrations of 1 to 100 μM (*p* < 0.001) (Fig. 2c). To examine which subtypes of P2R are involved in poly(I:C)-potentiated MUC5AC release via extracellular ATP, we first used P2X7R antagonists because P2X7R was recently implicated in the augmentation of various mediators after stimulation of TLRs [10]. A non-selective P2X7R antagonist, Brilliant Blue G (BBG), significantly inhibited poly(I:C)-potentiated MUC5AC release at 50 and 100 μM (*p* < 0.001) (Fig. 2d), but a specific P2X7R antagonist, A438079, did not at concentrations up to 100 μM (Fig. 2e). To account for this discrepancy, the effect of P2X7R silencing on poly(I:C)-induced MUC5AC release was evaluated. Although treatment with siRNA decreased the P2X7R protein, P2X7R knockdown did not affect poly(I:C)-augmented MUC5AC release (*p* < 0.05) (Fig. 3a and b). Next, we examined the involvement of P2Y2R in poly(I:C)-potentiated MUC5AC release because P2Y2R stimulation induced mucin release from secretory glands [11, 12]. Since there is no available selective inhibitor for P2Y2R, siRNA for P2Y2R was used to estimate the inhibitory effect on the release of MUC5AC. Treatment with P2Y2R siRNA suppressed P2Y2R protein expression (*p* < 0.05) (Fig. 3c) and significantly inhibited the poly(I:C)-potentiated MUC5AC release (*p* < 0.001) (Fig. 3d).

### Effect of P2R inhibition on EGFR ligand expression and release in poly(I:C)-treated NCI-H292 cells

**Fig. 4:**
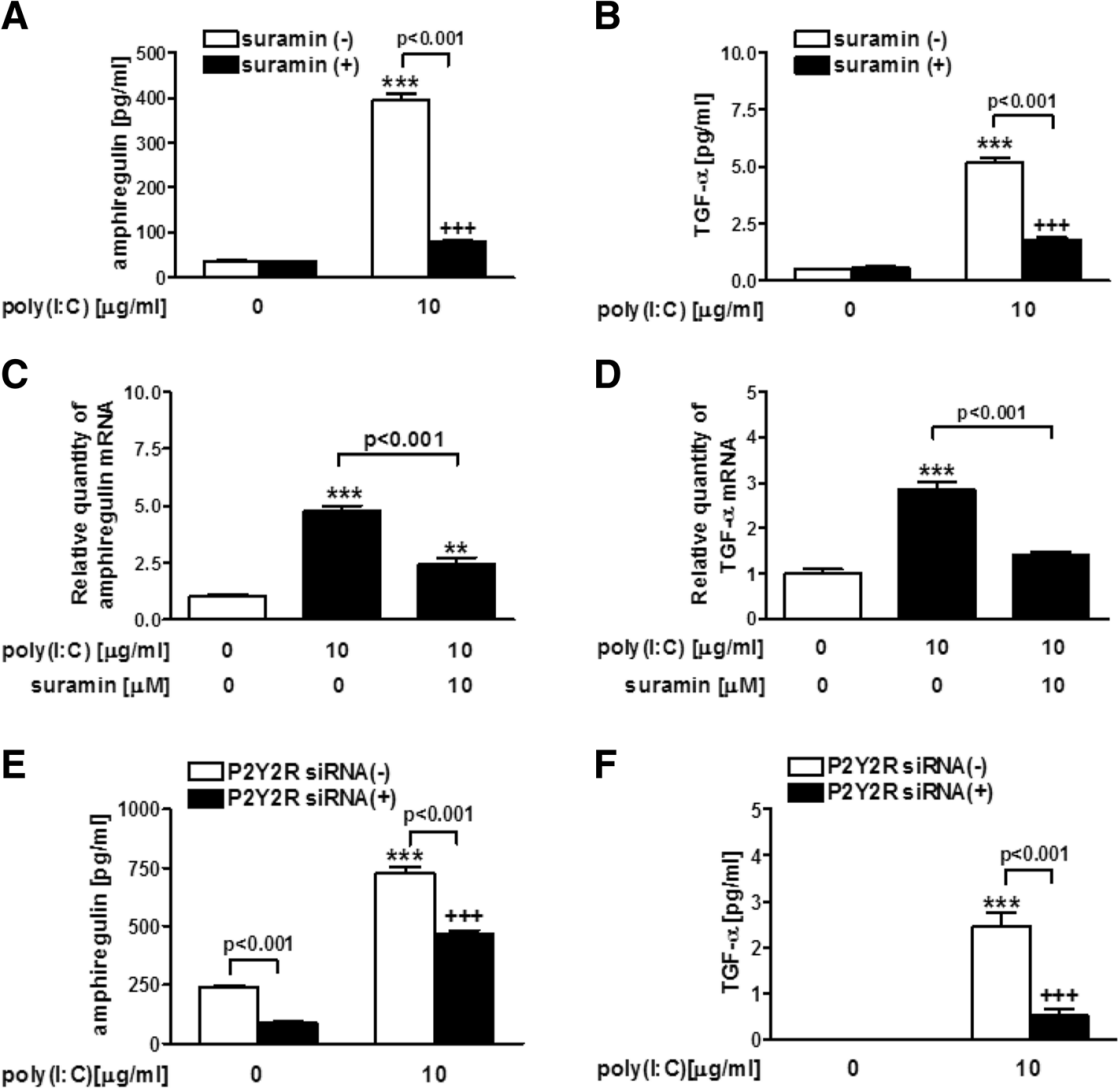
Effect of P2R antagonist on EGFR ligand release and expression in poly(I:C)-treated NCI-H292 cells. **a**-**d** Effect of a non-selective antagonist of P2R, suramin on the release and expression of EGFR ligands (amphiregulin and TGF-α). A 10 micromolar concentration of suramin was added 30 min before 10 μg/ml poly(I:C) treatment. After 24 h, whole cells and supernatants were harvested and assayed for the release of amphiregulin and TGF-α by ELISA and the expression of amphiregulin and TGF-α gene by Real-time PCR. **e**, **f** Effect of P2Y2R silencing with siRNA on the release of amphiregulin and TGF-α on poly(I:C)-treated cells. Cells were treated with P2Y2R silencing by siRNA prior to the treatment with 10 μg/ml poly(I:C). After 24 h, the supernatants were harvested and assayed for the release of amphiregulin and TGF-α. The data are expressed as the means ± SEM for three to four separate experiments. P2Y2R siRNA(-) siRNA denotes non-targeting siRNA treatment. **p < 0.01, ***p < 0.001 compared with the values of vehicle-treated control. ^+++^ *p* < 0.001 compared with the values of suramin or siRNA-treated control

The EGFR-ERK signaling pathways were reported to be involved in poly(I:C)-induced MUC5AC expression and release [21, 22]. To clarify whether P2R is involved in poly(I:C)-potentiated EGFR ligands, we investigate the effect of P2R antagonist and P2Y2R silencing with siRNA on the release and expression of EGFR ligands, amphiregulin and TGF-α in NCI-H292 cells after poly(I:C) stimulation. Treatment with 10 μM suramin significantly inhibited the release and expression of amphiregulin (*p* < 0.001) (Fig. 4a and c) and TGF-α (*p* < 0.001) (Fig. 4b and d). Treatment with P2Y2R siRNA significantly suppressed the release of amphiregulin (*p* < 0.001) (Fig. 4e) and TGF-α (*p* < 0.001) (Fig. 4f) in poly(I:C)-treated cells.

### Effect of P2R antagonist on the phosphorylation of EGFR and ERK in poly(I:C)-treated NCI-H292 cells

**Fig. 5:**
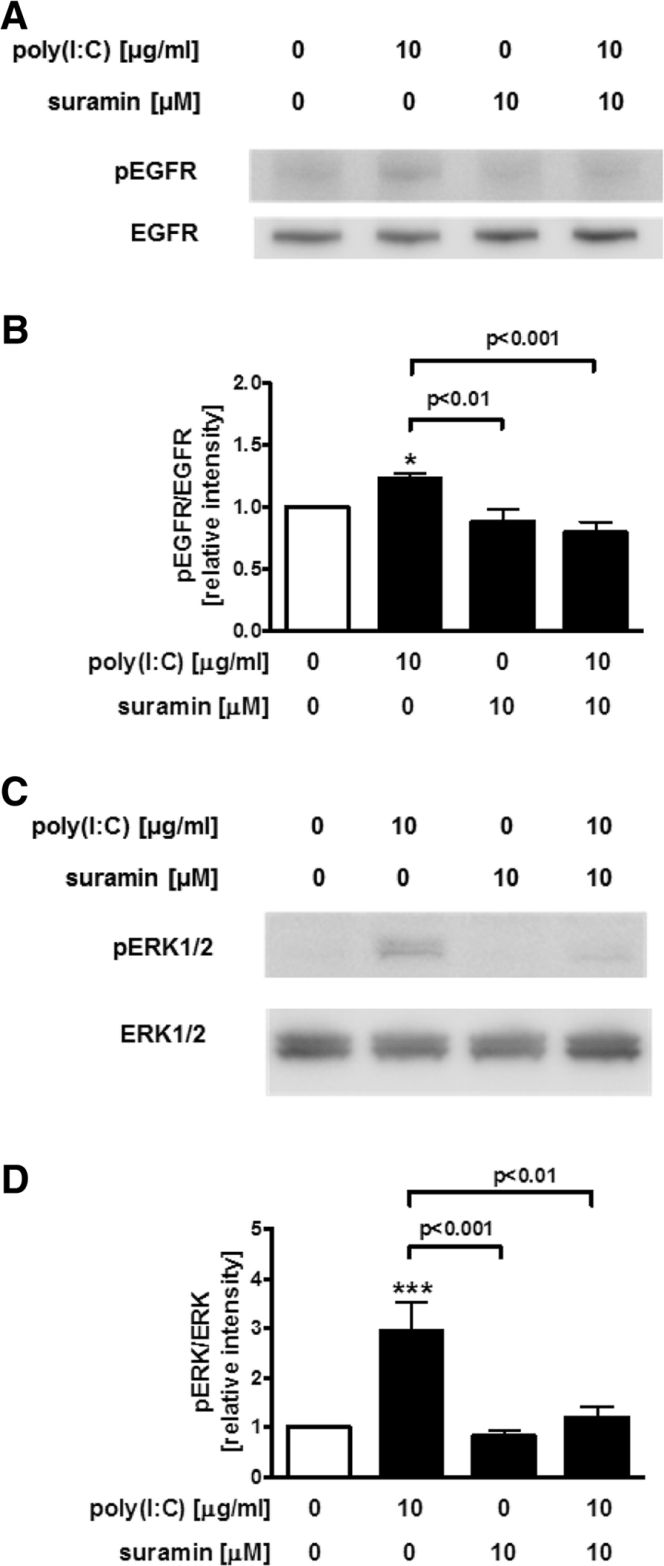
Effect of P2R antagonist on the phosphorylation of epidermal growth factor receptor (EGFR) and extracellular signal-regulated kinase (ERK) in poly(I:C)-treated NCI-H292 cells. Suramin was added 30 min before 10 μg/ml poly(I:C) treatment in NCI-H292 cells. After 4 h, whole cell lysates were obtained. **a**, **b** Phosphorylation of EGFR or ERK1/2 was evaluated with immunoblotting. Band intensity was assessed with densitometry. **c**, **d** Relative intensity was calculated by dividing the phosphorylated EGFR or ERK1/2 band intensity by the EGFR or ERK1/2 band intensity and the results were indicated as fold induction over control. The data are expressed as the mean ± SEM for three to four separate experiments. *p < 0.05, ***p < 0.001 compared with the values of vehicle-treated control

Next, we investigated the effect of P2R antagonist on EGFR and ERK1/2 phosphorylation after poly(I:C) stimulation. Because treatment with poly(I:C) significantly increased EGFR and ERK1/2 phosphorylation after 4 h in our previous study [22], we evaluated the effect of suramin 4 h after stimulation with poly(I:C). Treatment with 10 μM suramin significantly reduced the phosphorylation of EGFR (*p* < 0.001) (Fig. 5a and b) and ERK1/2 in poly(I:C)-treated NCI-H292 cells (*p* < 0.01) (Fig. 5c and d).

### Involvement of extracellular ATP on MUC5AC release in virus infected NCI-H292 cells

**Fig. 6:**
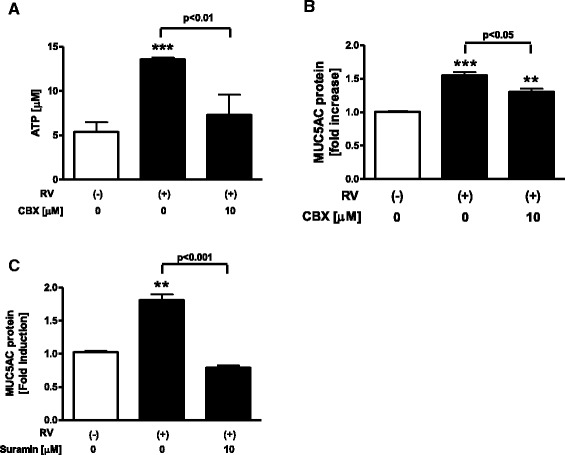
Involvement of extracellular ATP on MUC5AC release in viral-infected NCI-H292 cells. **a**, **b** Effect of a pannexin channel inhibitor, carbenoxolone (CBX), on the release of extracellular ATP (**a**) and MUC5AC (**b**) in RV14-infected NCI-H292 cells. Cells were treated with 10 μM CBX or vehicle 1 h prior to RV 14 infection. Cells were infected with RV 14 for 90 min and the virus was removed and replaced with medium. After 24 h or 48 h incubation, supernatants were harvested and assayed. Supernatants were assessed for the concentration of extracellular ATP by fluorometric assay and MUC5AC release by ELISA. **c** Effect of a non-selective antagonist of P2R, suramin, on MUC5AC release in RV 14-infected cells. Cells were treated with 10 μM suramin or vehicle 30 min prior to RV 14 infection. After 48 h RV 14 infection, the supernatants were harvested and assayed for MUC5AC release by ELISA. The data are expressed as the means ± SEM for three to four separate experiments. **p < 0.01, ***p < 0.001 compared with the values of vehicle-treated control

To confirm that the effects we observed with poly(I:C) could be replicated with live virus in NCI-H292 cells, we performed additional experiments using human rhinovirus, RV 14. RV 14 infection significantly increased the extracellular ATP and MUC5AC release (Fig. 6a and b) (ATP; 5.38 ± 1.13 vs 13.6 ± 0.25 μM at 24 h, *p* < 0.01, MUC5AC release; 1.55-fold increase at 48 h, *p* < 0.01). Pre-treatment with the pannexin channel inhibitor CBX at 10 μM reduced the amount of extracellular ATP and partially suppressed MUC5AC release from RV 14-infected cells (Fig. 6a and b). In addition, pre-treatment with the P2R antagonist suramin at 10 μM significantly reduced the release of MUC5AC from RV 14-infected cells (Fig. 6c). Titers of RV14 in the culture supernatants measured with endpoint methods using human embryonic fibroblasts [27] were 4.4 ± 0.2 Log TCID_50_ units/mL (mean ± SE, *n* = 5) at 48 h after infection. However, no virus was detected in the culture supernatants after infection with ultraviolet (UV)-irradiated RV14 as previously reported [27] (data not shown).

### Effect of pannexin channel inhibitor or P2R antagonist on the expression of MUC5AC in poly(I:C)-treated differentiated human bronchial epithelial cells from COPD patients

**Fig. 7:**
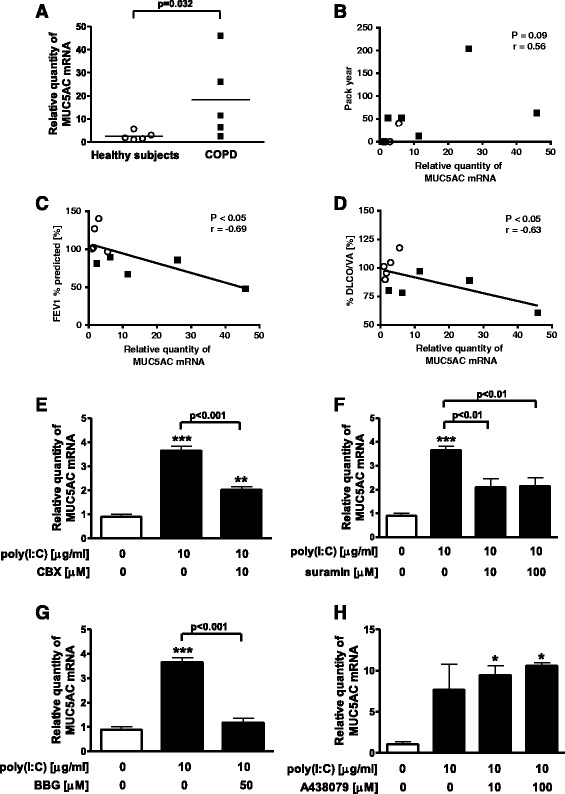
Effect of pannexin channel inhibitor or P2R antagonist on the expression of MUC5AC in poly(I:C)-treated differentiated human bronchial epithelial cells from COPD patients. **a** Expression of MUC5AC in poly(I:C)-treated HBECs from healthy subjects and COPD patients. HBECs were treated with 10 μg/ml poly(I:C). After 24 h, whole cells were harvested and assayed for the expression of MUC5AC gene by Real-time PCR. The data are expressed as the mean ± SEM of five healthy subjects or five COPD patients. **b**-**d** The relationship between the expression of MUC5AC in poly(I:C)-treated HBECs and smoking history (pack-years), the values of forced expiratory volume in 1 s (FEV_1_) %predicted, or carbonmonoxide diffusing capacity corrected for alveolar ventilation (DLCO/VA) %predicted. P-value and r were determined by Spearman’s correlation analysis in all subjects. Open circles (○) show healthy subjects and closed squares (■) show COPD patients. r is the correlation coefficient. **e**, **f** Effect of a pannexin channel inhibitor, carbenoxolone (CBX), or a P2R antagonist, suramin, on the expression of MUC5AC in poly(I:C)-treated HBECs from COPD patients. Cells were treated with CBX 1 h or suramin 30 min before 10 μg/ml poly(I:C) stimulation. After 24 h, whole cells were harvested and assayed for the expression of MUC5AC gene. The data is a representative of three independent experiments with three to five samples performed by using HBECs from three COPD patients, expressed as mean ± SEM. **p < 0.01, ***p < 0.001 compared with the values of control. **g**, **h** Effect of P2X7R antagonists on the expression of MUC5AC in poly(I:C)-treated HBECs from COPD patients. Various concentrations of a P2X7R antagonist, BBG, or a specific P2X7R antagonist, A438079, were added 30 min before 10 μg/ml poly(I:C) treatment. After 24 h, whole cells were harvested and assayed for the expression of MUC5AC gene. The data is a representative of three independent experiments with two to five samples performed by using HBECs from three COPD patients, expressed as mean ± SEM. *p < 0.05, **p < 0.01, ***p < 0.001 compared with the values of control

To confirm the involvement of pannexin channel and P2R in MUC5AC production after TLR3 stimulation in human bronchial epithelial cells (HBECs), we used differentiated HBECs, which mimic the in vivo features. To estimate the MUC5AC production in differentiated HBECs, we evaluated the expression of MUC5AC because the increase of MUC5AC expression was well correlated with that of MUC5AC release in NCI-H292 cells in our previous study [22]. Before this examination, we evaluated the expression of MUC5AC in differentiated HBECs between healthy subjects and age-matched COPD patients with poly(I:C) stimulation. After poly(I:C) stimulation, the expression of MUC5AC in the differentiated cells from COPD patients was significantly higher than those from healthy subjects (*p* = 0.032) (Fig. 7a). The values of the expression of MUC5AC in poly(I:C)-treated HBECs were inversely related with forced expiratory volume in 1 s (FEV1) % predicted (*r* = −0.69, *p* < 0.05) and carbon monoxide diffusing capacity corrected for the alveolar ventilation (DLCO/VA) %predicted (*r* = −0.63, *p* < 0.05), but not to the smoking history (pack-years) (*r* = 0.56, *p* = 0.09) (Fig. 7b-d). Concerning the expression of TLR3 in the differentiated HBECs after poly(I:C) stimulation, there was no difference between the healthy subjects and COPD patients (data not shown).

Next, we investigated the effect of pannexin channel inhibitor, CBX or the P2R antagonist, suramin on the poly(I:C)-potentiated expression of MUC5AC in differentiated HBECs from COPD patients. Treatment with 10 μM CBX and 10 μM and 100 μM suramin significantly suppressed the poly(I:C)-augmented expression of MUC5AC (*p* < 0.001 and *p* < 0.01) (Fig. 7e and f). In addition, we investigated the effect of P2X7R antagonist on the poly(I:C)-potentiated expression of MUC5AC in differentiated HBECs from COPD patients. A non-selective P2X7R antagonist, BBG significantly inhibited poly(I:C)-potentiated MUC5AC release at 50 μM (*p* < 0.001) (Fig. 7g), but a specific P2X7R antagonist, A438079, did not at concentrations of up to 100 μM (Fig. 7h).

### Cell viability

The effects of the inhibitors on cell viability were assessed with the 3-(4,5-dimethylthiazol-2-yl)-2,5-diphenyltetrazolium bromide (MTT) assay in NCI-H292 cells, and with the LDH assay in differentiated HBECs. Cell viability in the NCI-H292 cells was more than 89.7 % after the treatment with poly(I:C), ATP, rhinovirus infection and the inhibitors excluding CBX. Cell viability was 78.8 % at the highest dose of CBX after poly (I:C) treatment. In the differentiated HBECs, cell viability was more than 75.6 % after the treatment with poly(I:C) and the inhibitors.

## Discussion

**Fig. 8:**
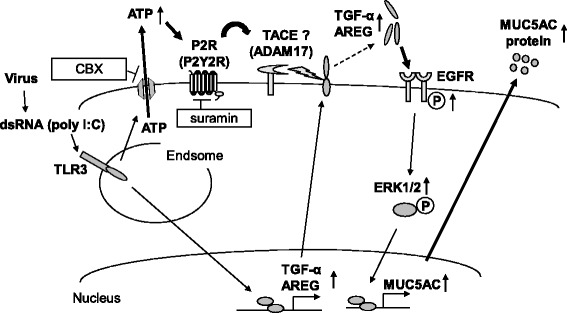
Schematic representation of the involvement of extracellular ATP on the release of MUC5AC induced by toll-like receptor 3 stimulation. In this graphic summary, the involvement of TACE activated by ATP is supported by reference 36 and the involvement of EGFR-ERK pathway in dsRNA-induced MUC5AC expression and release is also supported by reference 21 and 22. AREG: amphiregulin

In the present study, we demonstrated that a synthetic dsRNA analogue, poly(I:C), used as a TLR3 ligand to mimic viral infection, increased the amount of extracellular ATP and that this effect was reversed by a pannexin channel inhibitor, carbenoxolone (CBX). Stimulation with ATP induced the production and release of MUC5AC in human airway epithelial cells. In addition, we showed that pre-treatment with an inhibitor of P2R, suramin or P2Y2R siRNA, but not P2X7R siRNA significantly reduced the expression and release of MUC5AC and EGFR ligands, and the phosphorylation of EGFR and ERK in poly(I:C)-treated cells, suggesting that extracellular ATP is involved in dsRNA-induced MUC5AC production via P2R, especially P2Y2R (Fig. 8). These results were replicated with human rhinovirus and the inhibitory effects of CBX and suramin on poly(I:C)-potentiated MUC5AC expression were confirmed in differentiated human epithelial cells from COPD patients. These data suggest that viral-derived dsRNA induces the release of ATP via pannexin channel and that extracellular ATP is involved in the release of MUC5AC mainly via P2Y2R in an autocrine manner during viral-induced COPD exacerbation.

In the present study, we firstly demonstrated that dsRNA stimulation and viral infection increased the amounts of extracellular ATP via pannexin channel in NCI-H292 cells. Pannexin channel has been reported to be involved in the release of ATP from inflammatory cells and HBECs [7, 8]. Baxter M, et al demonstrated that cigarette smoke-bubbled medium induced the release of ATP from HBECs via pannexin channel and that the amount of extracellular ATP reached its peak at 3 h and reversed to the basal level at 24 h [7]. However, in the present study, the amount of extracellular ATP increased at 4 h and peaked at 16 h, which is inconsistent with the previous study. This discrepancy might have resulted from differences in the stimulation or in the experimental conditions including the type of cells. Another possibility is that the actual starting time of ATP release might be earlier than that of the net amount of ATP we measured because extracellular ATP is promptly decomposed by its metabolic enzyme, ectonucleotidases in the extracellular space [4]. In the current study, the inhibitory effect of CBX on the poly(I:C)-induced ATP release was partial, suggesting that other mechanisms might be involved in the poly(I:C)-induced ATP release, since various pathways, such as exocytosis, specific membrane transporters, or nonselective channels, have been also reported to be involved in the release of intracellular ATP to extracellular compartments [4, 29]. In the current study, it remains unclear what mechanisms mediate the activation of the pannexin channel. In previous reports, the involvement of P2X7R activation or hypotonic stress was reported to activate the pannexin channel [8, 9, 30]. However, the involvement of these mechanisms in the activation of the pannexin channel after dsRNA stimulation or RV 14 infection remains unclear. Further studies are needed to clarify these points.

In the present study, the potentiating effect of ATP on the release of MUC5AC in NCI-H292 cells was decreased at higher concentrations. This was possibly not likely due to toxicity, because the viability of the cells was not affected. This might be due to the existence of negative feedback regulation of MUC5AC release after a high dose of ATP, such as desensitization and down regulation of P2 receptors [31, 32].

The expression of P2Y1R, P2Y2R, P2Y4R, P2Y6R and P2XRs on the apical surface has been demonstrated in human airway epithelial cells [33, 34]. In the present study, we confirmed the expression of P2Y2R and P2X7R in NCI-H292 cells by western blot. A non-selective antagonist of P2R, suramin, and a non-selective P2X7R antagonist, Brilliant Blue G (BBG), significantly inhibited poly(I:C)- and RV 14-potentiated MUC5AC release, but a selective P2X7R antagonist A438079 did not. This discrepancy might have resulted from differences in the selectivity of these antagonists. P2X7R knockdown also did not inhibit the release of MUC5AC, suggesting that P2X7R is not involved in the poly(I:C)-induced MUC5AC release. However, since the efficacy of P2X7R knockdown was partial, it remains difficult to completely exclude the possible involvement of P2X7R. BBG is a non-selective P2X7R antagonist and the selectivity for human P2X7 (IC_50_ = 200 nM) is only 15 times higher than that for P2XRs, especially P2X4 (IC_50_ = 3.2 μM) [35]. At a 50 micromolar or higher concentration of BBG MUC5AC inhibition was observed, which might inhibit other P2XRs including P2X4. This suggests that there remains the possible involvement of other P2XRs including P2X4R in the poly(I:C) - and RV 14-potentiated MUC5AC release. On the other hand, there have been several reports showing the involvement of P2Y2R in mucin release from secretory glands [11, 12]. In the present study, P2Y2R knockdown significantly inhibited poly(I:C)-augmented MUC5AC release, which is consistent with previous studies. These data suggest that P2Y2R is mainly involved in the dsRNA-induced MUC5AC expression and release in NCI-H292 cells. However, the inhibitory effect of P2Y2R knockdown on poly(I:C)-induced MUC5AC release was only partial. This might be due to the low efficiency of uptake of the P2Y2R siRNA into the cells. As a limitation in this siRNA study, there remains the possible involvement of other P2 receptors in this mechanism because of the partial inhibitory effect of P2Y2R knockdown on poly(I:C)-induced MUC5AC release.

EGFR-ERK signaling pathways have been reported to be involved in poly(I:C)-induced MUC5AC expression and release in our and others’ previous studies [21, 22]. In the current study, we showed that the P2R antagonist suramin and a P2Y2R siRNA significantly suppressed the release of EGFR ligands (amphiregulin and TGF-α) in poly(I:C)-treated NCI-H292 cells. In addition, suramin significantly reduced the phosphorylation of EGFR and ERK1/2 in poly(I:C)-treated NCI-H292 cells. These results suggest that P2R, especially P2Y2R is involved in the poly(I:C)-induced MUC5AC release upstream of the EGFR-ERK pathway. Recently, Sham D, et al. reported that ATP activates an NADPH oxidase homolog, DUOX1, via P2Y2R leading to H_2_O_2_ production and that the H_2_O_2_ stimulates ADAM17, which is also called tumor necrosis factor-α converting enzyme (TACE) and is responsible for the shedding of EGFR pro-ligand and the activation of EGFR ligands [36]. This mechanism might be involved in the poly(I:C)-induced MUC5AC release. However, it remains unclear how P2Rs activate the EGFR- ERK pathway after poly(I:C) stimulation; further studies are needed to clarify this point.

The expression of MUC5AC in the airways of COPD patients has been reported to be increased compared to healthy subjects [37]. In the present study, we demonstrated, for the first time as far as we know, that MUC5AC expression in COPD patients was significantly greater than that in healthy subjects after dsRNA stimulation in differentiated HBECs under an air-liquid interface condition. In the current study, the values of MUC5AC expression in poly(I:C)-treated differentiated HBECs were inversely related to FEV1% predicted and DLCO/VA % predicted, which reflects damage to the alveolar capillary surface in patients with emphysema. This result suggests more mucus secretion in severe COPD during viral infections, which might lead to more frequent exacerbations of COPD [14–16]. Therefore, this result may support that COPD exacerbations become more frequent as the severity of COPD increases [38, 39].

Concerning the mechanisms in the augmented expression of MUC5AC in COPD patients, there might be several possibilities including the increased expression of TLR3 or the augmented response of the EGFR-ERK signal pathway. A previous report has shown that TLR3 expression was increased in the lung tissues or HBECs from COPD patients compared to those from non-smokers [40]. However, in the current study, there was no difference of the expression of TLR3 in the differentiated HBECs between healthy subjects and COPD patients in the limited number of samples. In our previous study, we showed that cigarette smoke, which is a major cause of COPD, augmented the response of EGFR-ERK signal pathway in NCI-H292 cells [22]. However, it remains unclear whether the augmentation exists in differentiated HBECs from COPD patients; further studies are needed to clarify this point. As a limitation of the present study, in differentiated HBECs, it is possible that sex differences and smoking had an influence on the results because HBECs were derived from 4 female healthy control subjects and 0 female COPD patients. In addition, only one of the healthy control subjects was an ex-smoker, whereas all 5 of the COPD patients were ex-smokers. In our previous study, we showed that cigarette smoke augmented poly(I:C)-stimulated MUC5AC mRNA in differentiated HBECs [22]. However, in the present study, there was no positive relation between smoking history and the values of MUC5AC expression after poly(I:C) stimulation. This discrepancy might be due to the small sample numbers. Further studies are needed to clarify these points.

In the differentiated HBECs from COPD patients, as well as in the NCI-H292 cells, we confirmed the inhibitory effect of pannexin channel inhibitor (CBX), non-selective P2R antagonist (suramin) and non-selective P2X7 antagonist (BBG), but not the selective P2X7 antagonist (A438079), on the poly(I:C)-potentiated MUC5AC expression. Seminario-Vidal, et al. previously reported that CBX inhibits hypotonic-induced ATP release via pannexin channel in differentiated HBECs [41]. In addition, the inhibitory effect of suramin on ATP analog-induced mucin secretion has been also shown in differentiated HBECs [12]. These results support the possible involvement of extracellular ATP in dsRNA-induced MUC5AC expression via P2R in differentiated HBECs.

## Conclusions

We demonstrated that dsRNA and viral infection induce the release of ATP via pannexin channel and that extracellular ATP is involved in the expression and release of MUC5AC via P2R, especially P2Y2R, in NCI-H292 cells and differentiated HBECs from COPD patients. These data suggest the involvement of extracellular ATP in viral-derived dsRNA-induced mucin expression and release mainly via P2Y2R in an autocrine manner during viral-induced COPD exacerbation. Modulation of this pathway could be a therapeutic target for viral-induced mucus hypersecretion in COPD exacerbations.

## Abbreviations

ALI: Air-Liquid Interface
ANOVA: One way analysis of variance
ATP: Adenosine-5′-triphosphate
BBG: Brilliant Blue G
CBX: Carbenoxolone
COPD: Chronic obstructive pulmonary disease
DLCO: Diffusing capacity of the lung for carbon monoxide
dsRNA: Double-stranded RNA
EGFR: Epidermal growth factor receptor
ERK: Extracellular signal-regulated kinase
FBS: Fetal bovine serum
FEV1: Forced expiratory volume in one second
FVC: Forced vital capacity
GAPDH: Glyceraldehyde-3-phosphate dehydrogenase
GOLD: The Global Initiative for Chronic Obstructive Lung Disease
HBECs: Human bronchial epithelial cells
P2Rs: P2 receptors
PDGF: Platelet-derived growth factor
poly(I,C): Polyinosine-polycytidylic acid
SEM: Standard error of the mean
siRNA: Small interfering RNA
TACE: Tumor necrosis factor-α converting enzyme
TGF-α: Transforming growth factor-α
TLRs: Toll-like receptors
VA: Alveolar volume
